# Pediatric provider perspectives and practices regarding health policy discussions with families: a mixed methods study

**DOI:** 10.1186/s12887-020-02238-y

**Published:** 2020-07-13

**Authors:** Aditi Vasan, Polina Krass, Leah Seifu, Talia A. Hitt, Nadir Ijaz, Leonela Villegas, Kathryn Pallegedara, Sindhu Pandurangi, Morgan Congdon, Beth Rezet, Chén C. Kenyon

**Affiliations:** 1 Department of Pediatrics, Children’s Hospital of Philadelphia, 3400 Civic Center Boulevard, Philadelphia, PA USA; 2 National Clinician Scholars Program, Perelman School of Medicine, University of Pennsylvania, Blockley Hall, 13th Floor, 423 Guardian Drive, Philadelphia, PA 19104 USA; 3 PolicyLab, Children’s Hospital of Philadelphia, 2716 South St. Roberts Center for Pediatric Research, 10th Floor, Philadelphia, PA USA; 4 Department of Pediatrics, Division of Critical Care Medicine, Columbia University, 630 West 168th Street, New York, NY USA; 5 Cincinnati Children’s Hospital Medical Center, Division of Gastroenterology, 3333 Burnet Ave, Cincinnati, OH USA; 6 Perelman School of Medicine, University of Pennsylvania, 3400 Civic Center Boulevard, Philadelphia, PA USA

**Keywords:** Advocacy, Health policy, Political engagement, Primary care

## Abstract

**Background:**

Advocacy regarding child health policy is a core tenet of pediatrics. Previous research has demonstrated that most pediatric providers believe collective advocacy and political involvement are essential aspects of their profession, but less is known about how pediatric providers engage with families about policy issues that impact child health. The objectives of this study were to examine providers’ perceptions and practices with regards to discussing health policy issues with families and to identify provider characteristics associated with having these discussions.

**Methods:**

In this cross-sectional mixed methods study, pediatric resident physicians, attending physcians, and nurse practitioners at primary care clinics within a large academic health system were surveyed to assess (1) perceived importance of, (2) frequency of, and (3) barriers to and facilitators of health policy discussions with families. Multivariable ordinal regression was used to determine provider characteristics (including demographics, practice location, and extent of civic engagement) associated with frequency of these discussions. A subset of providers participated in subsequent focus groups designed to help interpret quantitative findings.

**Results:**

The overall survey response rate was 155/394 (39%). The majority of respondents (76%) felt pediatricians should talk to families about health policy issues affecting children, but most providers (69%) reported never or rarely having these discussions. Factors associated with discussing policy issues included being an attending physician/nurse practitioner (OR 8.22, 95% CI 2.04–33.1) and urban practice setting (OR 3.85, 95% CI 1.03–14.3). Barriers included feeling uninformed about relevant issues and time constraints. In provider focus groups, four key themes emerged: (1) providers felt discussing policy issues would help inform and empower families; (2) providers frequently discussed social service programs, but rarely discussed policies governing these programs; (3) time constraints and concerns about partisan bias were a barrier to conversations; and (4) use of support staff and handouts with information about policy changes could help facilitate more frequent conversations.

**Conclusions:**

Pediatric providers felt it was important to talk to families about child health policy issues, but few providers reported having such conversations in practice. Primary care practices should consider incorporating workflow changes that promote family engagement in relevant health policy discussions.

## Introduction

Pediatricians have served as child health advocates since the field’s inception. Abraham Jacobi, one of the founders of pediatric medicine, famously stated, “every physician is by destiny a political being.” [1] This responsibility has been formalized by the American Academy of Pediatrics (AAP) in their policy statement on Poverty and Child Health, which recommends that pediatricians “advocate for public policies that support all children and mitigate the effects of poverty on child health,“ [2] and by the Accreditation Council for Graduate Medical Education (ACGME), which mandates that all pediatric residency programs provide residents with advocacy training [3].

Previous research has demonstrated that a majority of physicians believe collective advocacy and political involvement are important aspects of the medical profession and that most physicians, including pediatricians, have participated in these activities [4]. In a recent survey of pediatricians in the United States, the majority of respondents felt that their professional organization, the AAP, should engage in advocacy around government policies impacting child health, including policies related to income support, housing, education, and access to health care [5]. These studies show that pediatricians are motivated to engage in and support advocacy on behalf of their patients. However, less is known about these providers’ perceptions and practices in empowering children and families to advocate directly for themselves and their own communities.

Recent proposed policy changes have demonstrated the potential relevance of discussing health policy in a clinical setting. When funding for the Children’s Health Insurance Program (CHIP) lapsed in 2017 and early 2018, pediatric providers were encouraged by national child health organizations, including the AAP, to advocate for the program’s reauthorization [6]. Around the same time, two editorials written by internal medicine physicians called upon providers in their field to discuss policy changes related to health insurance coverage with their patients more directly [7, 8]. More recently, the AAP issued a statement emphasizing the potentially harmful chilling effects of the “public charge” rule in leading families to disenroll from or avoid applying for necessary health and social service programs. The AAP encouraged pediatricians caring for immigrant children to talk to their patients and families about the “public charge” rule and to explain to families that many government benefits, including CHIP and Medicaid for children under the age of 21, are still not considered in public charge determination [9].

We developed this study to better understand pediatric providers’ perspectives and behaviors when it comes to discussing policy issues like health insurance coverage and policy changes like the “public charge” rule with families as part of their clinical practice. Previous literature on this topic is limited to one published study examining provider perspectives on health policy conversations with patients through a survey administered to 36 internists [10]. Our study builds on this work both by specifically assessing pediatric providers’ perspectives and by utilizing an explanatory sequential mixed methods design, including an initial quantitative survey and subsequent focus groups designed to explore and gain deeper insights into survey responses.

Our aims were to (1) assess pediatric providers’ perspectives and practices in discussing health policy issues with patients and families, (2) examine provider characteristics associated with having these discussions, and (3) identify and understand providers’ perceived barriers to and facilitators of these discussions.

## Methods

### Study design and setting

This cross-sectional, explanatory sequential mixed-methods study of pediatric primary care providers including resident physicians, attending physicians, and nurse practitioners was conducted within a large, mid-Atlantic primary care practice-based research network of 31 primary care practices [11], which includes a pediatric residency program. This study was approved for exemption by the relevant Institutional Review Board.

### Provider survey

In the first part of this study, an electronic survey was distributed to all physicians within the pediatric residency program (*n* = 157) and all primary care providers within the care network (*n* = 246) between July 2018 and September 2018, with two subsequent reminder emails sent to each group. Respondents included resident physicians (physicians-in-training who practice in both the primary care and inpatient settings), nurse practitioners (advanced practice nurses who practice primary care independently), and attending physicians (physicians who have completed training and are responsible for both practicing primary care independently and supervising resident physicians). In the survey, child health policy was defined as “any aspect of local, state, or federal laws or regulations that may impact children’s health”, similar to the Centers for Disease Control definition [12].

### Quantitative outcomes

The survey was designed to assess two outcomes (1) providers’ perceptions of the importance of discussing health policy issues with families, and (2) providers’ self-reported frequency of these conversations. To assess perceived importance of policy discussions, providers were asked to rate their agreement with the statement, “Pediatricians should talk to families about current health policy issues affecting children,” using a 4-point Likert scale, where responses of “strongly agree” or “agree” were then classified as agreeing that these discussions were important.

To assess reported frequency of these conversations, providers were asked to indicate how frequently they discussed policy issues with families using a 4-point scale with options “never,” “rarely,” “sometimes,” and “always.” To identify provider factors associated with the practice of discussing policy issues with families, we operationalized provider frequency of these conversations as an ordinal dependent variable (with categories “never,” “rarely,” “sometimes,” and “always”) in our multivariable logistic regression model.

### Exposures and covariates

The survey assessed multiple covariates including level of training, years of experience, demographic characteristics, political affiliation, and civic and political engagement. Civic and political engagement were assessed in two ways: asking whether providers had voted in the most recent presidential and midterm elections and asking providers to both evaluate the importance of and report their recent participation in three categories of civic engagement, as initially described by Gruen et al. [4]. These categories were: collective advocacy (encouraging medical organizations to advocate for the public’s health), community participation (providing health-related expertise to community organizations), and political involvement (involvement in health policy related matters at the local, state or federal level), each assessed using 4-point Likert scales. Providers were categorized as “civic minded” if they rated the importance of civic engagement in each of these categories highly and as “civically engaged” if they reported taking part in any activity included in collective advocacy, community participation, or political involvement within the previous three years, consistent with the original study [4]. We hypothesized that providers with more clinical experience and providers who were more civically and politically engaged may report talking to families about health policy issues more frequently.

Providers were asked to select their most significant barriers to and facilitators of health policy discussions from a list generated through literature review and piloting of the survey instrument. The barriers listed included time constraints, discomfort with discussing policy issues, concerns that policy conversations would be perceived negatively, and concerns about perceived partisan bias. The facilitators listed included informational handouts regarding relevant policy issues and additional support staff to facilitate policy conversations. In both cases, providers also had the option to suggest additional barriers and facilitators not included in the provided list in the form of open-ended comments. These comments were subsequently reviewed, and barriers and facilitators that overlapped with existing categories were reclassified.

### Provider focus groups

In the second part of this study, we used our survey results to inform development of a focus group guide and then convened focus groups intended to help interpret and elaborate on our survey findings, consistent with an explanatory sequential mixed-methods approach with integration through building.

Six 30–45 min provider focus groups were held between November 2018 and March 2019. All providers who completed the survey were invited to participate, and we held the focus groups with a convenience sample of providers who were available at pre-designated times. Three focus groups included only resident physicians, while the other three included both attending physicians and nurse practitioners. A discussion guide was used for all focus groups (Appendix A2), with suggested questions focused on interpreting our quantitative results and obtaining a more in-depth understanding of providers’ perceived barriers to and facilitators of health policy discussions.

### Data analysis

Descriptive statistics were used to characterize providers who responded to the survey. Fisher’s exact tests were used to determine differences in perceptions and practices by provider level of training (resident physician versus attending physician/nurse practitioner). Multivariable ordinal logistic regression was used to examine variables associated with providers’ reported frequency of health policy discussions. Independent variables in this model included demographic characteristics (gender, race/ethnicity, and age), measures of political engagement (party affiliation, voting history, civic engagement score), and practice setting. All independent variables were dichotomized with the exception of age. Stratified analyses were conducted to assess for differences in associations by trainee status. Survey data was analyzed using STATA 15.1 (College Station, TX).

Focus groups were audio-recorded, transcribed, and analyzed using QSR International’s NVIVO12 software (Burlington, MA) using a modified grounded theory approach [13]. Transcripts were independently reviewed and coded by two researchers (PK and AV). The study team iteratively reviewed codes, identified emerging themes, and resolved any discrepancies through consensus. Initial codes used to generate these themes included perceived importance of policy discussions, frequency of policy discussions, barriers to discussing policy, and facilitators of policy discussions. Although resident physician and attending physician/nurse practitioner focus groups were held separately, all focus group discussions were grouped together in this qualitative analysis because similar themes emerged from the two subsets of providers.

## Results

### Participant characteristics

A total of 157 providers completed the survey, for an overall response rate of 39%. The response rate was 58% for resident physicians and 27% for attending physicians/nurse practitioners. Table 1 displays participant characteristics. Most respondents were female (76%) and identified as White (78%). The median age of respondents was 30 years. Seventy-six percent of providers (including all residents) practiced at one of three urban primary care practices, with the remainder practicing at suburban primary care sites. The majority of participants (75%) reported Democratic party affiliation.

**Table 1:** Characteristics of Study Participants

Characteristics	Number of participants (%) (***n*** = 157)
Gender	
Male	37 (24%)
Female	119 (76%)
Level of Training	
Resident Physician	91 (58%)
Attending Physician	55 (35%)
Nurse Practitioner	11 (7%)
Race/Ethnicity	
White	122 (78%)
Asian	16 (10%)
Hispanic	12 (8%)
African-American	6 (4%)
Practice Setting	
Urban	120 (76%)
Suburban	37 (24%)
Political affiliation	
Democrat	117 (75%)
Republican	7 (4%)
Independent	13 (8%)
Other/Prefer not to say	20 (13%)
Voting history	
Last midterm election	102 (65%)
Last presidential election	151 (96%)
Both elections	102 (65%)
Public participation	
Civic minded^a^	143 (91%)
Local community involvement	111 (71%)
Local, state or federal political involvement	100 (64%)
Organizational advocacy	129 (82%)
Civically engaged^b^	98 (62%)
Local community involvement	50 (32%)
Local, state or federal political involvement	67 (43%)
Organizational advocacy	46 (29%)

^a^Participants were defined as civic minded if the sum of scores of community participation, political involvement, and collective advocacy (range from 3 to 12) were greater than 10

^b^Participants were defined as civically engaged if they answered “yes” to taking part in any activity in community participation, political involvement, or collective advocacy in the past 3 years

Nearly all participants (96%) reported voting in the most recent presidential election, and 65% reported voting in the most recent midterm election. A significantly greater proportion of attending physicians/nurse practitioners reported voting in the most recent midterm election, as compared to resident physicians (82% vs. 61%, *p* < 0.001). Almost all providers (91%) were classified as “civic minded” and 98 providers (62%) were “civically engaged”.

A total of 28 providers, including 14 attending physicians, 11 residents, and 3 nurse practitioners participated in one of our six focus groups.

### Importance of health policy discussions

**Fig. 1:**
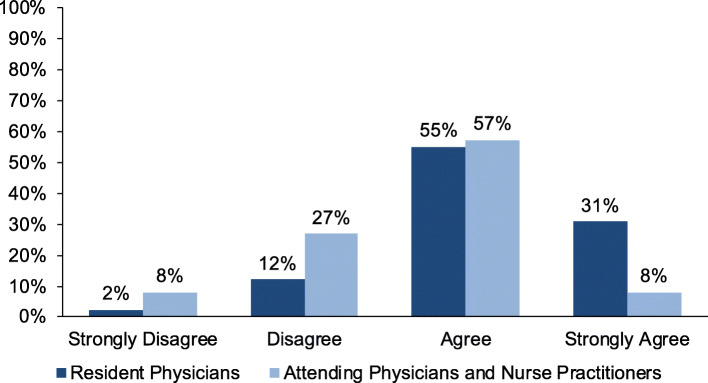
Provider Beliefs Regarding Health Policy Discussions with Families. Difference between resident physicians and attending physicians or nurse practitioners is statistically significant (*p* = 0.001)

Overall, the majority of survey respondents (78%) agreed that pediatricians should talk to families about health policy issues affecting children, including 86% of resident physicians as compared to 66% of attending physicians/nurse practitioners (Fig. 1, *p* = 0.001).

In focus groups, participants similarly agreed that it was important for pediatricians to discuss health policy issues with families (Table 2). Several providers felt that discussing relevant health policy issues was an important way for them to inform and empower their patients and families. One provider said, “I think we should be involved in helping to empower families to make their voices heard…the kids themselves don’t have a voice, you know, it’s us as their providers, keeping in mind their best interest, and their parents” (Resident Physician B).

### Frequency of health policy discussions

**Fig. 2:**
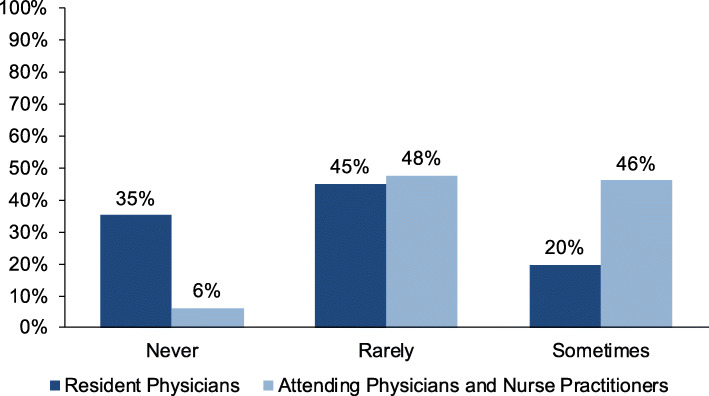
Providers’ Self-Reported Frequency of Health Policy Discussions with Families. No participants selected “always.” Difference between resident physicians and attending physicians or nurse practitioners is statistically significant (*p* < 0.001)

Although the majority of providers felt health policy discussions with families were important, few reported having these discussions in practice, with 69% of providers reporting never or rarely discussing policy issues. A significantly greater proportion of resident physicians (80%) as compared to attending physicians/nurse practitioners (54%) reported never or rarely having these conversations (Fig. 2, *p* < 0.001).

In focus groups, some providers described more frequently discussing policy issues that seemed directly relevant to a patient’s current medical problems (Table 2). One provider said, “If there’s something that’s immediately related to something [families] bring up or that you elicit in the interview, then I think that could be really an effective time to talk about this” (Resident Physician D). Other providers said they frequently discussed individual social services such as nutrition and education programs with patients, but did not usually frame these as broader policy issues. “One of the things I find a natural way to bring up is nutrition programs [like] WIC [and] SNAP...I don’t normally get to the next step of ‘you should support those things’ or ‘you should think about your elected officials and whether they support those things’” (Attending Physician E).

In our multivariable regression (Table 3), factors associated with increased frequency of health policy discussions included being an attending physician or nurse practitioner, as compared to being a resident physician (OR 8.2, 95% CI 2.0–33.1) and practicing in an urban setting as compared to a suburban setting (OR 3.8, 95% CI 1.0–14.3). In analyses stratified by provider type (resident versus attending/nurse practitioner), voting in the previous midterm election was associated with increased odds of having health policy discussions for resident physicians (OR 2.8, 95% CI 1.1–7.1), and urban practice setting was associated with increased odds of having these discussions for attending physicians/nurse practitioners (OR 9.2, 95% CI 1.7–49.2).

**Table 2:** Importance and frequency of policy discussions: representative quotations and major themes elicited from focus group analysis

Themes		Representative Quotations
Perceived importance of policy discussions	Discussing relevant policy issues allows providers to inform and empower families	“Anything you can do to lend your voice, and particularly empower your patients to lend their voices to these issues is really important.” – Attending Physician A “If [patient] testimony is what’s necessary to change things*…*[providers] should be in a position to educate people and get them involved, since we’re their contact with the healthcare system.” – Resident Physician A “I think we should be involved in helping to empower families to make their voices heard… the kids themselves don’t have a voice, you know, it’s us as their providers, keeping in mind their best interest, and their parents.” – Resident Physician B
Current frequency of policy discussions	Clinicians more frequently discuss timely issues or issues that are directly related to provision of medical care	“I think it was useful around the time of the election … when we were also trying to register families [to vote], to use that as a current event that could help you talk about policy issues.” – Resident Physician C “If there’s something that’s immediately related to something [families] bring up or that you elicit in the interview, then I think that could be really an effective time to talk about this. “ – Resident Physician D
	Clinicians frequently discuss social service programs with families, but do not often discuss the policies governing these programs	“When I talk to my families, I’m not talking broad policy things, I’m more assessing their situation - Are the kids in preschool or Head Start?… Do they have food insecurity?” – Attending Physician B “I probably feel a little more comfortable talking about … resources, and to make sure that they’re aware of what resources they’re eligible for and if not, how to get them.” – Resident Physician E

**Table 3:** Multivariable Model Results Assessing Associations between Provider Characteristics and Frequency of Health Policy Discussions with Families.*

	OR (95% CI)		
	All providers	Residents	Attending Physicians and Nurse Practitioners
Provider experience*	8.22 (2.04–33.1)^a^	–	–
Gender	0.61 (0.27–1.38)	0.48 (0.19–1.24)	2.36 (0.40–13.7)
Non-white race /ethnicity	1.95 (0.67–5.65)	1.32 (0.38–4.61)	4.97 (0.39–63.5)
Age (y)	1.02 (0.96–1.08)	0.91 (0.71–1.15)	1.06 (0.98–1.15)
Democatic party affiliation	1.68 (0.72–3.95)	2.47 (0.82–7.41)	0.6 (0.12–2.95)
Urban practice setting	3.85 (1.03–14.3)^b^	0.93 (0.03–30.0)	9.22 (1.72–49.2)^d^
Voting history**	1.78 (0.82–3.84)	2.79 (1.10–7.08)^c^	0.29 (0.04–2.25)
Civic minded	1.68 (0.45–6.29)	1.54 (0.13–18.9)	0.89 (0.14–5.6)
Civically engaged	1.57 (0.77–3.21)	1.23 (0.52–2.9)	2.84 (0.66–12.1)

All independent variables are dichotomized apart from age

*Defined as being an Attending Physician or Nurse Practitioner, as compared to a Resident. **Defined as a history of voting in both the most recent midterm and most resident presidential election.

^a^*p* = 0.003

^b^*p* = 0.044

^c^*p* = 0.031

^d^*p* = 0.009

### Barriers to health policy discussions

**Fig. 3:**
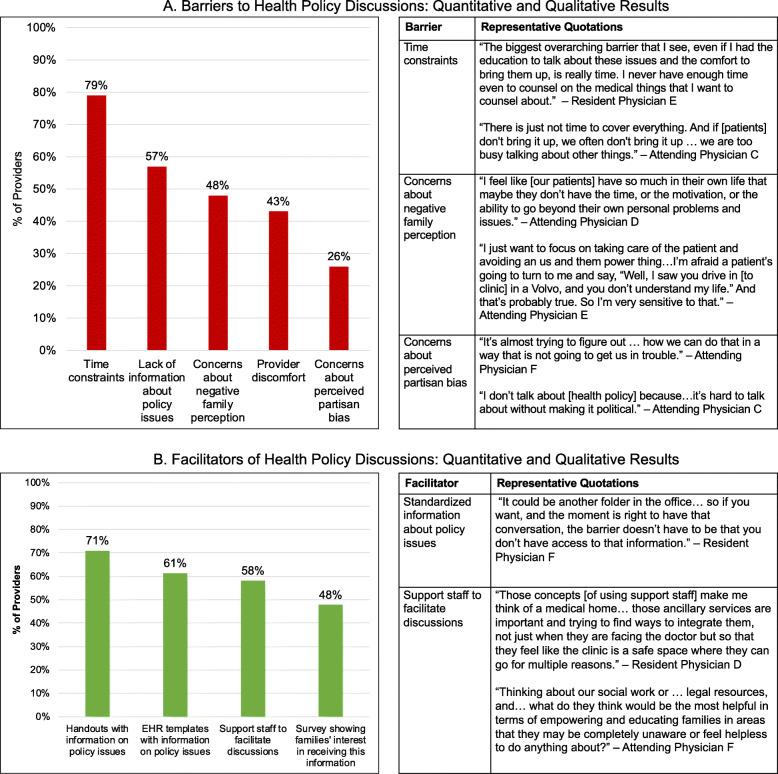
**a** Barriers to Health Policy Discussions: Quantitative and Qualitative Results. Time constraints were the most commonly reported barrier to having health policy discussions in quantitative analysis and represented a salient barrier in qualitative analysis. **b** Facilitators of Health Policy Discussions: Quantitative and Qualitative Results. Standardized information about policy issues and workflow changes involving support staff were frequently discussed facilitators for having health policy discussions in quantitative analysis and were commonly discussed and emphasized in quantitative analysis

Survey participants identified several key barriers to health policy discussions, including time constraints (79% of respondents), lack of knowledge about policy issues (57%), concerns about negative family perception (48%), and provider discomfort with discussing policy issues (43%) (Fig. 3A). Participants reported a total of 15 unique barriers to policy discussions in the survey.

In focus groups, the most salient barriers to health policy discussions identified by participants were time constraints, concerns about family perception, and concerns about perceived partisan bias. Several participants worried that patients’ families may perceive discussions about health policy in the clinical setting negatively. One physician stated, “I don’t talk about [health policy] because…it’s hard to talk about without making it political” (Attending Physician C). Focus group participants also raised concerns about how race, class, and power differentials between providers and patients might complicate these discussions. One provider said, “I’m afraid a patient’s going to turn to me and say, “Well, I saw you drive in [to clinic] in a Volvo, and you don’t understand my life.” And that’s probably true. So I’m very sensitive to that” (Attending Physician E).

### Facilitators of health policy discussions

In our survey, providers identified informational handouts (71% of respondents), electronic health record-based templates with health policy information (61%), and involvement of support staff (58%) as interventions that could help facilitate more frequent policy discussions (Fig. 3B). Survey participants reported a total of 10 unique facilitators of policy discussions.

Focus group participants similarly felt that information about policy issues available to providers and families, including written materials explaining relevant health policy issues and talking points that could be used when discussing these issues, could facilitate discussions. One provider remarked, “It could be another folder in the office…so if you want, and the moment is right to have that conversation, the barrier doesn’t have to be that you don’t have access to information” (Resident Physician F). Participants also recommended utilizing support staff, such as social workers or volunteers from community organizations focused on political engagement, to hold these discussions with families. As one participant explained, “Those concepts [of using support staff] make me think of a medical home… those ancillary services are important and [we should be] trying to find ways to integrate them…so that [families] feel like the clinic is a safe space where they can go for multiple reasons” (Resident Physician D).

## Discussion

To our knowledge, this study is the first investigation of pediatric provider perspectives and practices in discussing health policy topics with patients and families. We found a substantial disconnect between provider beliefs regarding the importance of these conversations and the frequency with which they reported having these discussions in practice. Factors associated with discussing health policy issues more frequently included increased provider experience and practicing in an urban setting.

In our sample, 78% of providers agreed that pediatricians should talk to patients about health policy issues, yet 69% reported never or rarely having these conversations in practice. These results are similar to those of the one prior survey examining policy discussions among internal medicine physicians, in which 83% of respondents felt it was appropriate to remind patients to vote and 42% felt it was appropriate to discuss politically oriented health care issues in clinic, but only 42% reported initiating a discussion about voting and only 17% reported initiating a discussion about another politically oriented health care issue in their clinical practice [10].

This discordance between providers’ perceptions of the importance of policy discussions and their self-reported frequency of discussing policy issues is consistent with a larger body of behavioral research. There are several behavioral models that have been used to predict volitional action, including the Theory of Planned Behavior [14, 15], which has been used to explain discrepancies between physicians’ ideal behavior and reported action in a wide range of settings, including adherence to clinical guidelines [16–19] and counseling on specific topics [20, 21]. In these cases, differences between perception and practice are influenced by physician intention, social normalization, and physicians’ perceived ability to perform the action. This model suggests that even if physicians believe a new intervention or approach is important, they will be unlikely to adopt it unless they believe it is both feasible and accepted among their peers.

Our observed disconnect between perceptions and practices was more prominent for resident physicians, with 86% agreeing that pediatricians should discuss policy issues, but 80% reporting never or rarely having these discussions. This finding was consistent with our multivariable model findings, which demonstrated that attending physicians and nurse practitioners had increased odds of having policy discussions as compared to resident physicians. These results could be partly explained by the barriers elucidated by respondents in our survey and focus groups. Resident physicians may face more significant time constraints than their non-trainee colleagues due to their relative inefficiency. They may also be less likely to have longitudinal relationships with families that could build trust and provide a basis for potentially sensitive discussions.

We hypothesized that civic engagement and civic mindedness may have been associated with increased rates of health policy discussions but found no association between these characteristics and frequency of policy discussions in our multivariable model. This may have been partially because the majority of our respondents, both residents and attendings, were classified as both civic minded and civically engaged. There may also have been collinearity between these measures and other independent variables included in the analysis, such as voting history or political affiliation.

Our multivariable regression showed an association between urban practice setting and increased frequency of policy discussions. Providers at these urban practices serve a predominantly low-income, Medicaid insured population and therefore may have perceived policy issues related to government programs and benefits as more directly relevant to their patient population. Providers choosing to practice in these settings may also place a higher priority on understanding and influencing the impact of health policies on their patients’ health.

Our findings suggest that pediatric practices should consider workflow changes that promote a broader framework of child and family engagement in important health policy discussions in the clinic. Several providers in our study expressed a desire to have resources supporting health policy discussions integrated into clinic visits, for example, through informational handouts available to families in the waiting room or offered to families by clinic support staff. Interventions such as these may also empower patients to initiate discussions about health policy with their providers, which may mitigate providers’ concerns about patients perceiving these conversations negatively. Clinics could also partner with community-based organizations focused on promoting political engagement to hold co-located voter registration drives or informational sessions about relevant policy issues, allowing families to obtain this information before or after bringing their children to clinic visits without taking up valuable time during a clinical encounter. Our findings suggest that incorporating these interventions in resident primary care clinics may be particularly beneficial.

There are a number of limitations to our study. Our overall survey response rate of 39% was relatively low, although comparable to prior published studies of survey research involving pediatricians, which reported response rates ranging from 29 to 46% [22–24]. Variable response rates to our questionnaire may have skewed the sample if participants who viewed health policy discussions more favorably were more likely to respond. Further, a majority of respondents identified as civically engaged, potentially making them more likely to support policy discussions than physicians who were less civically engaged. Despite this, few respondents reported routinely having these conversations in practice, suggesting that our findings may represent a conservative estimate.

Our data was gathered from a single network of academic primary care clinics, with an associated office of government affairs and a relatively low-income, high-need patient population, particularly at the urban clinic sites. In addition, the residents in this sample all trained in a program with a robust longitudinal advocacy curriculum. It may therefore be difficult to generalize our results to other practice settings. While we attempted to define the term “health policy” specifically in our questionnaire and focus groups, it is possible that providers interpreted this term more broadly as any conversation with families about government benefits programs for which they may be eligible, which may have increased the number of participants identifying these conversations as important or reporting having these conversations in practice.

Additionally, we used single survey items, rather than validated survey instruments, to measure our primary outcome variables of interest, perceived importance of screening and self-reported frequency of screening. To our knowledge, there are no existing validated instruments or composite scores to assess these constructs. Future surveys focused on patient-provider conversations about health policy issues may help to validate both our selected survey questions and our findings.

Our focus groups represent a convenience sample of providers who were available during pre-selected focus group times, rather than with a purposefully selected subset of providers. This may have resulted in selection bias and may have made our focus group sample less representative of the overall sample of providers who completed the survey.

Lastly, and importantly, while this study investigates provider attitudes and practices, it does not include patient or family perspectives on having health policy discussions in clinical settings. Additional research should explore family perspectives in order to create and implement interventions aimed at facilitating these discussions in ways that are efficient, meaningful, relevant, and acceptable to families.

## Conclusion

We found that the majority of pediatric primary care providers in our sample believed it was important to talk to families about health policy issues impacting children. However, few providers reported having these conversations in practice, with provider experience and practice in an urban setting associated with an increased frequency of policy discussions. There may be an opportunity for pediatric primary care practices to partner with community-based organizations to empower patients and families and create targeted informational materials focused on health policy issues that could facilitate these important conversations.

## Data Availability

The datasets used and/or analyzed during the current study are available from the corresponding author on reasonable request.

## Appendix

### Survey Questionnaire

In this survey, we are assessing [1] provider attitudes towards talking to families about policy issues relevant to children’s health and [2] provider attitudes toward advocating on their patients’ behalf.

In this survey, “policy issue” refers to an aspect of local, state, or federal laws or regulations that may impact children’s health. For example, a discussion of “gun-related policy” may involve the current gun-related laws and regulations, any proposed changes to these laws and regulations, and how an individual may become involved in advocacy related to these laws and regulations (by voting, rallying, petitioning, etc.). This is in contrast to family-specific screening (e.g. for food insecurity or poverty) and patient-specific anticipatory guidance (e.g. counseling about gun safety).

Please answer honestly. Your survey answers will be completely anonymous. This survey should take 5–10 min to complete.

**Part 1: Discussing policy issues with families.**

**1. Do you believe that pediatricians should encourage parents and age-eligible patients to vote?**

- Yes
- No
- Unsure

**2. Do you think believe that pediatricians should provide families with information about how to contact their local/state/federal representatives?**

- Yes
- No
- Unsure

**3. Please rate your agreement with the following statement: “I believe that pediatricians should talk to families about current health policy issues affecting children”**

(4 options: Strongly Disagree, Disagree, Agree, Strongly Agree)

**3a. For each of the specific policy issues below, please rate your agreement with the following statement:** “**I believe that pediatricians should talk to families about _________”:**

(4 options for each: Strongly Disagree, Disagree, Agree, Strongly Agree)

- Immigration policy.

- Gun-related policy.

- Health insurance coverage and access to care.

- Price and access to medications.

- LGBTQ/Transgender health policy (including policies surrounding access to care and policies relating to discrimination).

- Parental leave / childcare-related policy.

- Policies related to food assistance programs (SNAP, WIC, etc.)

- Policies related to early childhood education (Early Intervention, Head Start, Universal Pre-K, etc.)

- Policies related to education for school-aged children (eg. charter schools).

- Vaccination-related policy.

**4. How often do you currently talk to families about health policy issues affecting children?**

(4 options: Never (0–25% of the time)/Rarely (25–50% of the time)/Sometimes (50–75% of the time)/Always (More than 75% of the time))

**4a. How often do you currently talk to families about the following issues?**

(4 options: Never (0–25% of the time)/Rarely (25–50% of the time)/Sometimes (50–75% of the time)/Always (More than 75% of the time))

- Immigration policy.

- Gun-related policy.

- Health insurance coverage and access to care.

- Price and access to medications.

- LGBTQ/Transgender health policy (including policies surrounding access to care and policies relating to discrimination).

- Parental leave / childcare-related policy.

- Policies related to food assistance programs (SNAP, WIC, etc.)

- Policies related to early childhood education (Early Intervention, Head Start, Universal Pre-K, etc.)

- Policies related to education for school-aged children (eg. charter schools).

- Vaccination-related policy.

**5. What do you consider the major barriers to talking to families about these issues? (Select all that apply)**

- I don’t think we should talk to families about health policy issues
- I don’t have time to talk to families about these issues
- I don’t feel comfortable talking to families about these issues
- I worry that families will perceive conversations about health policy negatively
- I worry that my institution / organization will not approve of me discussing policy with patients
- I worry that these conversations will not be beneficial to patients and families
- I don’t have enough information about these issues
- Other: _____________

**6. What do you think would help overcome these barriers? (check all that apply)**

- Informational handouts regarding relevant political issues
- Templates in the electronic medical record related to relevant political issues
- Survey showing a patient/family’s level of interest in political information
- Having another member of the care team (SW, medical student, volunteer from an external organization) available to talk to interested families about relevant policy issues
- I don’t think it is appropriate to talk about these issues
- I don’t think my institution / organization will allow me to talk about these issues / give out this information
- Other: ______________

**7. What suggestions do you have about how we can more effectively talk to families about relevant health policy issues?**

(Free text)

**Part 2: Physician public roles and advocacy.**

**8. How important is it for physicians to provide health-related expertise to local community-organizations (eg. School boards, parent-teacher organizations, athletic teams, and local media)?**

(4 options: Not at all important/Not very important/Somewhat important/Very important)

**9. In the past 3 years, have you provided health-related expertise to local community organizations?**

- Yes.

- No.

**10. How important is it for physicians to be politically involved (other than voting) in health-related matters at the local, state, or national level?**

(4 options: Not at all important/Not very important/Somewhat important/Very important)

**11. In the past 3 years, have you been politically active (other than voting) on a local health care issue?**

- Yes.

- No.

**12. How important is it for physicians to encourage medical organizations to advocate for the public’s health?**

(4 options: Not at all important/Not very important/Somewhat important/Very important)

**13. In the past 3 years, have you encouraged your professional society to address a public health or policy issue that is not primarily concerned with physician welfare?**

- Yes.

- No.

**Part 3: Provider characteristics.**

**14. What is your current provider role?**

- Resident Physician
- Attending Physician
- Nurse Practitioner

**15. How many years have you been in practice since graduating from your medical school / nurse practitioner program?**

- < 5 years.

- 5-15 years.

- > 15 years.

**16. What is your main location of primary care practice?**

**17. What is your gender?**

- Male.

- Female.

- Other (free text):

**18. What is your ethnicity?**

- White / Caucasian.

- Black / African American.

- Hispanic.

- Asian or Pacific Islander.

- American Indian or Alaskan Native.

- Other (free text).

**19. What is your age?**

(Free text)

**20. What is your political affiliation?**

- Democrat
- Republican
- Libertarian
- Green
- Independent
- Other: _________
- Prefer not to say

**21. Did you vote in the most recent presidential election (2016)?**

- Yes
- No
- Prefer not to say

**22. Did you vote in the most recent congressional midterm election (2014)?**

- Yes
- No
- Prefer not to say

### Focus Group Guide

At the start of each focus group, we shared a written summary of our key survey findings with all participants. We then facilitated the focus groups using the suggested questions below:

Interpreting Survey Results.

1. What do you think about these results? What do you find most interesting? What do you find most surprising?

Importance of Advocacy and Policy Discussions.

*2. Do you think it is important for pediatricians to advocate on behalf of their patients? Why or why not?*

*3. Which health policy issues do you think are most important for pediatricians to bring up with their patients? Why do you think these issues are important?*

Barriers to and Facilitators of Policy Discussions.

*4. What do you think are the major barriers to talking to patients and families about health policy issues?*

*5. What do you think we could do to help providers more effectively talk to families about relevant health policy issues?*

*6. When it comes to talking to families about health policy issues, who do you think should be leading these discussions? (Eg. Physicians, nurses, social workers, medical assistants,* etc.*)*

*7. Is there anything else you would like to share with our team?*

## Abbreviations

AAP: American Academy of Pediatrics
ACGME: Accreditation Council for Graduate Medical Education
CHIP: Children’s Health Insurance Program
SNAP: Supplemental Nutrition Assistance Program
WIC: Special Supplemental Nutrition Program for Women, Infants, and Children
